# High Hospital-related Costs at the End-of-life in Patients With Multiple Myeloma: A Single-center Study

**DOI:** 10.1097/HS9.0000000000000913

**Published:** 2023-05-26

**Authors:** Christine Bennink, Hans Westgeest, Daan Schoonen, Femke Boersen, Pieter Sonneveld, Jan Hazelzet, Hedwig Blommestein, Marjolein van der Klift

**Affiliations:** 1 Department of Oncology and Hematology Breda, Amphia Hospital, Breda, The Netherlands; 2 Department of Hematology, Erasmus University Medical Center, Rotterdam, The Netherlands; 3 Department of Finance and Control/Business Intelligence Centre, Amphia Hospital, Breda, The Netherlands; 4 Department of Public Health, Erasmus University Medical Center, Rotterdam, The Netherlands; 5 Erasmus School of Health Policy and Management, Erasmus University, Rotterdam, The Netherlands

Due to prolonged survival and increasing treatment costs per patient, cancer treatment imposes an increasing burden on total healthcare expenditures. Healthcare expenditures in cancer will approximately increase from 5.6 billion euros in 2015 to 61 billion in 2060 in the Netherlands, of which 49 billion will be hospital costs.^1^ Previous research showed that treatment costs in cancer patients are highest in the first and last year of life.^2,3^ Health economics research demonstrated that proximity to death is a better predictor of increasing health expenditures than age, especially in cancer.^4,5^ Furthermore, aggressive treatment and higher costs of treatment at the end of life are associated with worse quality of life.^6–8^ Therefore, critically reviewing the use of intensive treatment at the end of life may provide valuable insights to improve the quality of life while reducing costs.

Although there is an increasing interest in end-of-life care in cancer patients, research on the costs of end-of-life care in hematological malignancies is limited. Patients with hematological malignancies, and multiple myeloma (MM) patients in particular, are more likely to undergo intensive hospital care compared with other cancer patients in the last month before death.^9–13^ Nevertheless, analyses addressing the burden and costs of end-of-life care in MM are lacking. Given the limited research on the use and costs of end-of-life care,^14^ we aimed to describe the hospital-related care in the last month before death in patients diagnosed with MM and to provide a comparison with other malignancies in our hospital.

In this single-site study, anonymized data from electronic health records were used. All MM patients deceased between 2017 and 2021 were included in our analyses. Hospital care activities were defined as all clinical and outpatient diagnostic, treatment (anticancer treatments excluded), follow-up, and aftercare activities by medical professionals performed in the Dutch hospital setting and were obtained from claims data, which are standardized throughout the Netherlands.

We used standardized cost estimates for the year 2021 of Amphia Hospital, which corresponded with costs in comparable nonacademic Dutch teaching hospitals (Suppl. Table S1). The costs of anticancer treatments, that is, medication treatments to stop the progression of cancer, were analyzed separately. Furthermore, subanalyses were performed by age, using the cutoff between old age and very old age of 80 years as is used by the World Health Organization,^15^ and by time since diagnosis, in which diagnosis could mean time of diagnosis or time of start of oncological treatment (after initial diagnosis by other medical departments). The total costs presented in this study are accumulated over 5 years (2017–2021) and mean costs per patient were calculated over the total group of deceased patients. To put the outcomes of the MM group in perspective, a cohort with reference data from patients with other malignancies (including 500 patients with hematological malignancies other than MM) was collected similarly. These patients were diagnosed and treated in Amphia Hospital with at least 1 hospital care activity in the last year before death (to only include patients who were recently actively treated or received follow-up for malignant disease) (Suppl. Table S2). Descriptive statistics for end-of-life care, defined as costs of hospital-related care in the last 30 days before death, were performed for the MM and the reference group. Because the comparison of groups was primarily intended to interpret the MM data, no inferential statistical analyses were performed.^16^

Between 2017 and 2021, a total of 131 deceased patients with MM were identified, with a median age at death of 76. In total 44 (33.6%) patients had anticancer treatments and 106 (81%) patients had hospital care activities in the last 30 days before death. In the reference group, 4841 deceased patients were identified with a median age at death of 73 years. In total 733 (15.1%) received anticancer treatments and 3582 (74%) patients had hospital care activities in the last 30 days before death (Suppl. Table S3).

The mean costs per patient of anticancer treatment were €1614 per patient in MM in the last 30 days before death between 2017 and 2021, which was higher than in the reference group, €452 per patient (Figure 1A). Also, the proportion of MM patients receiving anticancer treatment was higher than in the reference group (33.6% versus 15.1%). The higher costs per patient of anticancer treatments in MM may be caused by the fact that MM treatments are more expensive. The higher proportion of patients treated with anticancer treatment may be caused by sudden health deteriorations leading to death in actively treated patients. The mean costs per patient of hospital care activities in MM patients were €8367 per patient, which was considerably higher than in the reference group (€5414/patient) (Figure 1) and also higher than the mean anticancer treatment costs per patient. The total accumulated costs of anticancer treatment and hospital care activities in the last 30 days of life of patients with MM were €1,307,546 in 2017–2021 (see Suppl. Table S4 for more details on total costs).

**Figure 1.**
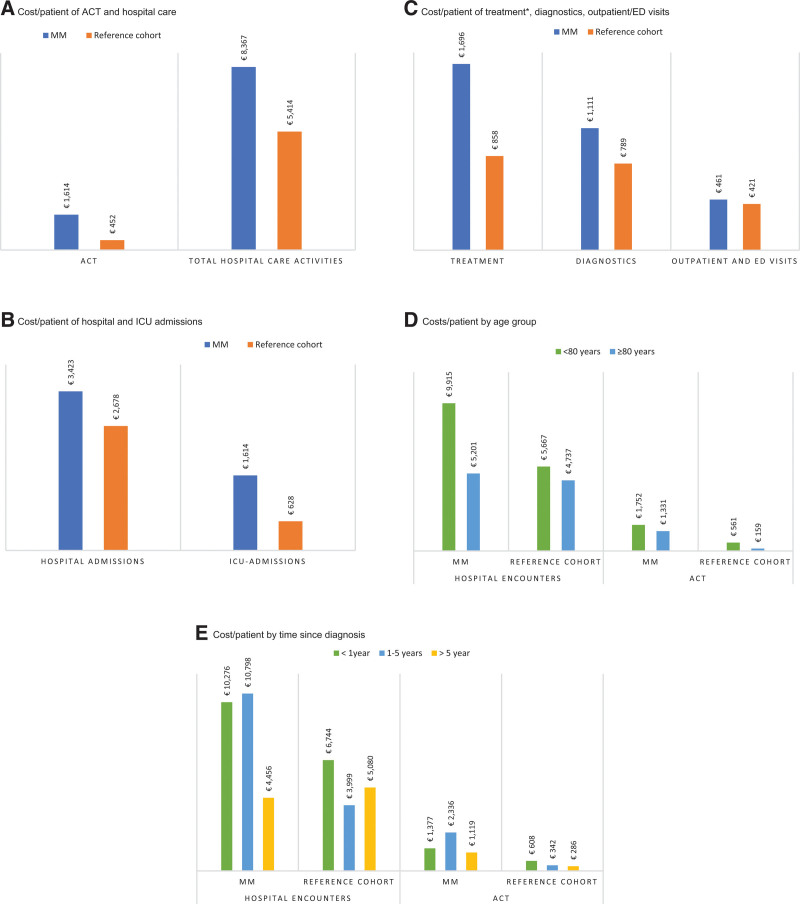
**Costs per patients 30 days before death in MM (N=131) and the reference group (N=4841).** (A) Cost per patient of ACT and hospital care. (B) Cost per patient of hospital and ICU admissions. (C) Cost per patient of treatment*, diagnostics, outpatient/ED visits. (D) Costs per patient by age group. (E) Cost per patient by time since diagnosis. *ACT excluded. The scales in figures (A)–(E) are different. MM = multiple myeloma; ACT = anticancer treatment; ICU = intensive care unit; ED = emergency department.

Analyses by type of hospital activity showed that hospital admissions in the last 30 days before death were observed in 57% (N=75) of the MM patients and caused the highest total end-of-life costs and €3424 per patient. Intensive care unit (ICU) admissions caused €1666 per patient (N=17, 13.0%). In the reference cohort, the proportion of patients with hospital and ICU admissions was lower (49% and 6.9%, respectively), and also mean costs per patient were lower (€2678 and €628, respectively), which means that relatively more MM patients were admitted to the hospital and ICU, with longer length of stay, compared with the reference group (Figure 1B).

Costs of treatments, which included (blood)transfusion therapy, dialysis, surgery, paramedical, and rehabilitation treatments (excluding anticancer treatments), in the last 30 days before death were observed in 65% (N=85) MM patients, with mean treatment costs per patient of €1696 per patient of which costs of blood transfusions and dialysis were the largest cost components (Suppl. Table S4). These (supportive) treatment costs were higher in MM than in the reference cohort and were equivalent to the costs of anticancer treatments in MM (Figure 1C).

Furthermore, diagnostics costs (N=92), including radiology and laboratory tests, were €1111 per patient, and outpatient and emergency department consultations costs were €461 per patient (N=90), which were both higher than in the reference group (Figure 1C).

Subanalysis by age groups showed that hospital care activities and anticancer treatment costs in the last 30 days before death were lower in MM patients ≥80 years (N=43) compared with younger MM patients (N=88). Hospital care activity costs and anticancer treatment costs were also considerably higher in MM patients <80 years compared with patients <80 in the reference group (Figure 1D).

In the subanalysis by time since diagnosis, we found €10,276 per patient hospital care activity costs in the last 30 days before death in MM patients who died within 1 year after diagnosis (N=39) and €10,798 per patient in MM patients who died between 1 and 5 years after diagnosis (N=45). In MM patients who survived >5 years after diagnosis, the total costs of hospital care activities were considerably lower at €4456 per patient (N=47). End-of-life anticancer treatment costs in MM patients based on the time since diagnosis showed the following: €1377 per patient in patients who died within 1 year, €2336 per patient in patients who died between 1 and 5 years, and €1119 per patient in patients who lived >5 years after diagnosis. The median survival since diagnosis in the MM group was 1039 days (2.9 years). In the reference group, all costs per subgroup were lower except in patients with >5 years since diagnosis (hospital care activity costs €5080 per patient), and the median survival time was 432 days (1.2 years) (Figure 1E).

The percentages of patients receiving intensive end-of-life treatment and costs per patient at the end of life are higher in MM compared with patients with other malignancies. This may be explained by the fact that more treatment lines are available in MM compared with other malignancies, which may explain the higher proportion of MM patients being treated near the end of life. Furthermore, unforeseen health deteriorations caused by infections or other adverse events leading to unexpected death are more common in MM. Also, MM patients are more often in need of (intensive) supportive treatments such as blood transfusions and dialysis treatments, compared with patients with other malignancies (Figure 2A and 2B).

**Figure 2.**
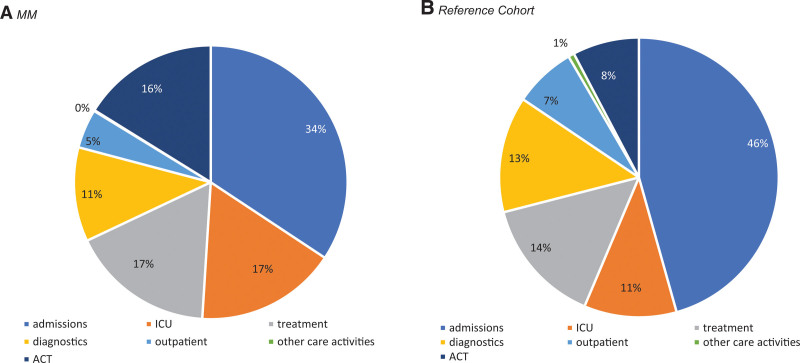
**Type of costs in the last 30 days before death in percentages of total costs including anticancer treatments.** (A) MM. (B) Reference cohort. MM = multiple myeloma; ACT = anticancer treatment; ICU = intensive care unit.

This study aimed to contribute to the scarce research on end-of-life costs of hematological malignancies and particularly in MM. However, the generalizability of the findings needs to be addressed with caution due to the small sample size. Data collection and analyses were relatively easy to perform and repeat with updated data. Nevertheless, hospital, hospice, or home care policies may differ between countries, which should be considered when comparing these outcomes to findings in other countries. Also, the high costs of new anticancer treatments versus off-patent medications need to be considered when monitoring end-of-life costs over time. Further research preferably in a larger population of MM patients, addressing the effect of end-of-life care on both quality of life and costs is necessary.

In conclusion, costs per patient in the last 30 days of life were higher in MM patients compared with patients with other malignancies, predominantly caused by hospitalizations and high costs of ICU admissions, especially in patients deceased within 5 years from diagnosis. Supportive treatment costs, primarily caused by blood transfusions and dialysis, were equal to the costs of anticancer treatment in MM patients’ last 30 days of life. More awareness about the high intensity and potential burden of treatment at the end of life in MM may improve the quality and efficiency of care, although further research to validate our results in other settings is necessary.

## AUTHOR CONTRIBUTIONS

CB, MK, and HW presented the idea. All authors actively participated in the study design. CB, DS, and FB executed the data capture. CB, DS, FB, MK, HW, and HB performed the analyses and drafted the article. All authors critically revised the article and approved the final version.

## DISCLOSURES

PS: Received Research grants from Amgen, Celgene, Janssen, Skyline Dx. Honoraria from Amgen, Celgene, Janssen, Karyopharm, Seagen, Chairman of European Myeloma Network, Co-chairman of HOVON Myeloma Working Group. HW: travel expenses Astellas and Ipsen; Honoraria Astellas, Roche, Merck. HB: Reports consulting or advisory role for Pfizer (paid to institute) and research funding from BMS-Celgene (paid to institute). All the other authors have no conflicts of interest to disclose.

## SOURCES OF FUNDING

The authors declare no sources of funding for this manuscript.

## Supplementary Material


