# Supplementary material for: High Hospital-related Costs at the End-of-life in Patients With Multiple Myeloma: A Single-center Study
Source: Hemasphere. 2023 May 26;7(6):e913. doi: 10.1097/HS9.0000000000000913 (PMC10256370; doi:10.1097/HS9.0000000000000913)
Supplement: Supplementary file 1 [file hs9-7-e913-s001.docx]

Supplementary Digital Content: Table 1

Supplementary Table 1: Estimate of costs/unit by group of care activity

*MM: Multiple Myeloma, ICU: Intensive Care Unit; non-ACT treatment: treatments other than anti-cancer treatments; ED: Emergency Department*
