# Supplementary material for: High Hospital-related Costs at the End-of-life in Patients With Multiple Myeloma: A Single-center Study
Source: Hemasphere. 2023 May 26;7(6):e913. doi: 10.1097/HS9.0000000000000913 (PMC10256370; doi:10.1097/HS9.0000000000000913)
Supplement: Supplementary file 2 [file hs9-7-e913-s002.docx]

Supplementary Table 2: Malignancies based on diagnosis treatment combination codes included in the analyses

|  | **DTC** | **DTC** |
| --- | --- | --- |
| **Breast cancer** | **Surgery** | **Oncology** |
| breast cancer | not included | 811 |
| **Uro-oncology** | **Urology** |  |
| kidney tumor | 10 | 834 |
| urethra tumor | 20 | 833 |
| bladder tumor | 30 | 833 |
| testis | not included | 831 |
| prostaat | not included | 832 |
| **Lungcancer** | **Pulmonology** | **Oncology** |
| NSCLC | 1303 | 622 |
| SCLC | 1304 | 621 |
| mesothelioma | 1305 | 314 |
| mediastinal tumor | 1306 |  |
| other pulmonary tumors | 1307 | 629 |
| **Dermato-oncology** | **Dermatology** | **Oncology** |
| melanoma | 350 | 842 |
| **Gynaecology** | **Gynaecology** | **Oncology** |
| ovarium | M16 | 821 |
| cervix | M13 | 822 |
| endometrium | M14 | 823 |
| vulva | M11 |  |
| **Hematology** | **Haematology** | **NA** |
| Hodgkin lymphoma | 751 |  |
| N-H lymphoma low grade | 752 |  |
| N-H lymphoma interm/ H grade | 753 |  |
| multiple myeloma* | 754 |  |
| ALL | 756 |  |
| CLL | 757 |  |
| AML | 761 |  |
| MDS | 762/763 |  |
| CML | 771 |  |
| **Gastro-enterology** | **Surgery** | **Oncology** |
| oesophagus | 319 | 904 |
| gall bladder | 331 |  |
| pancreas | 332 | 964 |
| colon | 333 | 927 |
| sigmoid | 334 | 927 |
| rectum | 335 | 927 |
| liver | 367 | 955 |
| stomach | 346 | 904 |
| other GE malignancies | 349 | 979 |

*DTC: Diagnosis Treatment Code*
