# Supplementary material for: High Hospital-related Costs at the End-of-life in Patients With Multiple Myeloma: A Single-center Study
Source: Hemasphere. 2023 May 26;7(6):e913. doi: 10.1097/HS9.0000000000000913 (PMC10256370; doi:10.1097/HS9.0000000000000913)
Supplement: Supplementary file 3 [file hs9-7-e913-s003.docx]

Supplementary Table 3: Patient characteristics

| **All patients 2017-2021** | MM  N= /(%) [min-max] | Reference Cohort  N= /(%) [min-max] |
| --- | --- | --- |
| Nr deceased | 131 | 4841 |
| Nr deceased with hospital care activities | 106 (81%) | 3582 (74%) |
| Nr deceased with ACT | 44 (33.6%) | 733 (15.1%) |
| Gender (male) | 89 (67.9%) | 2712 (56.0%) |
| Median age | 76 [44-93] | 73 [20-99] |
| Age group (≥80 years) | 43 (32.8%) | 1315 (27.2%) |

*MM: Multiple Myeloma, (%) percentage of total N deceased; ACT: Anti-Cancer Treatment*
