# Supplementary material for: High Hospital-related Costs at the End-of-life in Patients With Multiple Myeloma: A Single-center Study
Source: Hemasphere. 2023 May 26;7(6):e913. doi: 10.1097/HS9.0000000000000913 (PMC10256370; doi:10.1097/HS9.0000000000000913)
Supplement: Supplementary file 4 [file hs9-7-e913-s004.docx]

Supplementary Table 4: Total costs and costs per year of End-of-Life Care

|  | **MM (N-131)** | | **Reference group (N=4841)** | |
| --- | --- | --- | --- | --- |
|  | **total costs 2017-2022** | **mean cost/year** | **total costs 2017-2022** | **mean cost/year** |
| **Total end-of-life costs** | € 1,307,546 | € 261,509 | € 28,397,343 | € 5,679,469 |
| *** Total costs anti-cancer treatment** | € 211,411 | € 42,282 | € 2,187,800 | € 437,560 |
| ***Total costs hospital care activities** | € 1,096,135 | € 219,227 | € 26,209,543 | € 5,241,909 |
| **Admissions** | € 448,478 | € 89,696 | € 12,963,145 | € 2,592,629 |
| **ICU** | € 218,259 | € 43,652 | € 3,041,703 | € 608,341 |
| **Treatment** | € 222,169 | € 44,434 | € 3,497,935 | € 699,587 |
| *** Blood** | € 80,432 | € 16,086 | € 1,003,431 | € 200,686 |
| *** Dialysis** | € 36,443 | € 7,289 | € 110,782 | € 22,156 |
| **Diagnostics** | € 145,593 | € 29,119 | € 3,817,450 | € 763,490 |
| **ED and outpatient visits** | € 60,359 | € 12,072 | € 2,036,299 | € 407,260 |

*MM: Multiple Myeloma; ICU: Intensive Care Unit; ED: Emergency Department*
